# High prevalence of *Rickettsia* spp. in ticks from wild hedgehogs rather than domestic bovine in Jiangsu province, Eastern China

**DOI:** 10.3389/fcimb.2022.954785

**Published:** 2022-07-26

**Authors:** Yong Qi, Lele Ai, Jun Jiao, Junhu Wang, Deping Wu, Pengcheng Wang, Guoyu Zhang, Yong Qin, Cheng Hu, Ruichen Lv, Nianhong Lu, Changqiang Zhu, Yingqing Mao, Rui Qi, Yuexi Li, Weilong Tan

**Affiliations:** ^1^ Huadong Research Institute for Medicine and Biotechniques, Nanjing, China; ^2^ Nanjing Bioengineering (Gene) Technology Center for Medicines, Nanjing, China; ^3^ State Key Laboratory of Pathogen and Biosecurity, Beijing Institute of Microbiology and Epidemiology, Beijing, China; ^4^ Huaian Medical District of Jinling Hospital, Medical School of Nanjing University, Huaian, China; ^5^ 901^st^ Hospital, Hefei, China; ^6^ Xuyi County Hospital of Chinese Medicine, Huaian, China; ^7^ The 907^th^Hospital of Chinese PLA, Nanping, China; ^8^ Army Medical University, Shijiazhuang, China

**Keywords:** spotted fever group Rickettsia, tick, hedgehog, Rickettsia heilongjiangensis, Candidatus Rickettsia principis, Candidatus Rickettsia jingxinensis

## Abstract

**Background:**

Spotted fever group *Rickettsia* (SFGR), containing various pathogenic *Rickettsia* spp., poses remarkable negative influences to public health by causing various severe or mild diseases. Information regarding prevalence of SFGR in ticks in Jiangsu province, Eastern China, is still limited and needs urgent investigations.

**Methods:**

Hedgehog- and bovine-attached ticks were collected from Jiangsu province, Eastern China. DNA of individual ticks was extracted for nested polymerase chain reaction amplifications targeting *gltA*, 16S ribosomal RNA (*rrs*), *ompA*, *ompB*, and *sca4* genes following with sequencing. SFGR-specific IgG antibodies in sera of local donators were evaluated using ELISA.

**Results:**

Overall, 144 (83.2%) of the 173 ticks from hedgehogs and 2 (1.2%) of the 168 ticks from bovine were positive for one of the three identified *Rickettsia* spp., with significant difference between the two groups (*P* = 3.6e-52). *Candidatus* Rickettsia principis (9; 5.2%) and *R. heilongjiangensis* (135; 78.0%) were detected in *Haemaphysalis flava* rather than in *H. longicornis* ticks from hedgehogs. *R. heilongjiangensis* (1; 0.6%) and *Candidatus* R. jingxinensis (or *Candidatus* R. longicornii) (1; 0.6%) were identified in *H. longicornis* and *Rhipicephalus microplus* ticks from bovine, respectively. Phylogenetic analysis indicated *Candidatus* R. jingxinensis belonged to *R. japonica* subgroup, whereas *Candidatus* R. principis belonged to a novel subgroup. Higher serological prevalence of spotted fever and SFGR-specific IgG antibody level in humans were observed around the investigated area than in urban areas, without significant difference.

**Conclusion:**

*Candidatus* R. principis and *Candidatus* R. jingxinensis were identified in Jiangsu province, Eastern China, and fully genetically characterized for the first time. The higher prevalence of SFGR in hedgehog-attached ticks as well as the higher SFGR-specific IgG antibody level and seropositive rate in humans around the investigated area suggested that more attention should be paid to SFGR. This pathogen is usually transmitted or harbored by wild animals and ticks. This study provides important epidemiological data for both physicians and public health officers in developing early prevention and control strategies against potential *Rickettsia* infections and in the preparation of suitable testing and treatment needs for rickettsiosis in the endemic areas.

## Introduction

Ticks are the second most important vectors of pathogens (only to mosquitoes), causing diseases in both animals and humans and economic losses in livestock husbandry. They can also transmit a variety of pathogenic organisms, including viruses, bacteria, and protozoa (Yu et al., 2016; Huang et al., 2021; Song et al., 2021). In them, *Rickettsiae* are worldwide distributed and cause mild, severe, or even life-threatening Rickettsial diseases (Parola et al., 2013). At least 34 species in genus *Rickettsia* are classified into four groups: the spotted fever group (SFG), the typhus group, the ancestral group, and the transitional group, in which the SFG *Rickettsia* (SFGR) contains the largest number of *Rickettsia* spp. and is divided into four subgroups: *Rickettsia rickettsii*, *R. japonica*, *R. massiliae*, and *R. tamurae*, respectively (Diop et al., 2020; Wang et al., 2021). Nowadays, novel pathogenic or non-pathogenic *Rickettsia* spp. are still continuously been identified (Wang et al., 2021). In China, more than 10 species of SFGR have been detected, some of which cause severe human rickettsioses (Wang et al., 2021), indicating their potential threat to public health.

SFGR is mainly maintained and transmitted by ticks (Merhej and Raoult, 2011; Parola et al., 2013; Wang et al., 2021). Significant diversity of SFGR exists among different tick species, the ticks’ associated vertebrate reservoirs, and geographic distributions, which determines the risk area of tick-borne diseases (Parola and Raoult, 2001; Merhej et al., 2014). Therefore, it is of great significance for public health to clarify the local distribution of different tick species and their harboring SFGR (Wang et al., 2021).

**Figure 1:**
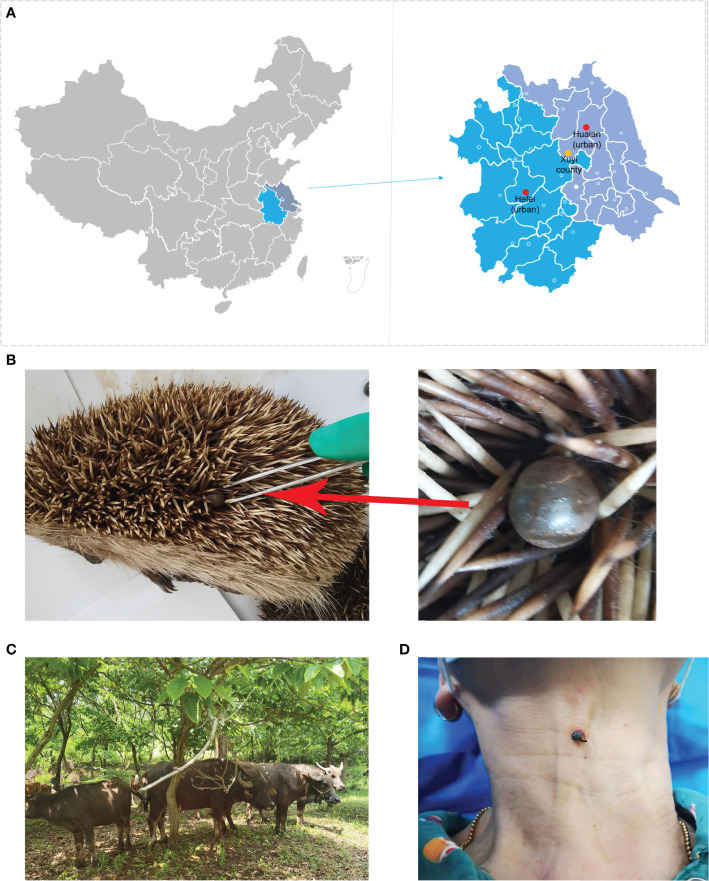
Sample collection sites and the observed tick hosts. **(A)** Locations of tick (yellow dot) and sera (red and yellow dots) collection sites; **(B)** a parasitic tick collected from an already dead hedgehog; **(C)** bovine hosts for tick collections; **(D)** a farmer bitten by a tick observed during our sample collection.

Jiangsu province, as a coastal province with warm and humid climate, low and flat terrain, and numerous rivers and lakes, provides a perfect ecological basis for the tick survival and reproduction. Tick-bitten patients were typically observed among farmers, stock raisers, and gardeners in this area (**Figure 1D**, a farmer bitten by a tick observed during our sample collection). A high incidence of tick-borne diseases has been reported in the area, such as severe fever with thrombocytopenia syndrome (SFTS) caused by SFTS virus and scrub typhus caused by *Orientia tsutsugamushi* (Liang et al., 2014; Hu et al., 2017; Hu et al., 2018; Lu et al., 2019; Zhang et al., 2019). However, the species of SFGR that ticks harbor or the role of ticks in the circulation of SFGR in Jiangsu province, Eastern China, is still not clear. In Yancheng city, Jiangsu province, *R. monacensis* was identified in a patient, *R. heilongjiangensis* and *R. japonica* were detected in *Apodemus agrarius* mice, whereas *R. japonica* was detected in *Haemaphysalis longicornis* ticks (Lu et al., 2019). The present study determined the prevalence of SFGR in ticks from wild hedgehogs and domestic bovine, clarified their molecular characteristics, and evaluated the threats posed by these SFGR to public health in Jiangsu province, Eastern China. The results provide useful epidemiological data that could be crucial for both physicians and public health officers to prevent and control potential *Rickettsia* infections.

## Materials and methods

### Sample collection and DNA extraction

The ticks used in the present study have been described in our previous study (Qi et al., 2022). Briefly, 341 ticks, including 173 adults from 32 wild hedgehogs (*Erinaceus amurensis*) and 168 adults from 30 domestic bovine (including 18 *Bubalus bubalis* buffalo, eight *Bos taurus* cattle, and four Chinese Holstein cows), were collected from May 2019 to October 2020 in rural area (E 118°29’6”, N 32°43’55”) in Xuyi county of Huaian city, Jiangsu Province, China (**Figures 1A–C**) (Qi et al., 2022). Four to 10 ticks were randomly sampled from each animal and preliminarily identified by their morphological features before they were washed and homogenized as described previously (Jiao et al., 2021a). Genomic DNA from individual ticks was extracted using a DNeasy Blood & Tissue Kit (Qiagen, Germany) as per the manufacturer’s instructions.

A total of 791 sera samples were collected from hospitals, including 323 samples collected in April 2022 from Xuyi County Hospital of Chinese Medicine around the tick collection site in the Xuyi county of Huaian city, 268 samples collected in October 2021 from Huaian Medical District of Jinling Hospital in the urban area of Huaian city, and 200 samples collected in November 2021 from the 901st Hospital in the urban area of Hefei city. The sera were used for routine hospital tests previously without additional purposeful collection. The locations were indicated in **Figure 1A**.

The use of human and animal samples was reviewed and approved by the Ethics Committee of Huadong Research Institute for Medicine and Biotechniques. The blood of donators was drawn for routine blood testing in the hospitals and randomly selected for this study. Written informed consent was obtained from the blood donators and bovine owners for the tick collection from their animals in this study. Information of the donators was deidentified.

### PCR amplification and sequencing

The species of the ticks were identified by the sequences of the mitochondrial 16S ribosomal RNA gene as previous description (Liu et al., 2016).

For *Rickettsia*-positive sample screening, nested PCR amplifications targeting a short partial sequence of *gltA* gene about 380 bp were conducted as elsewhere described (Jiao et al., 2021a). Briefly, each PCR amplification reaction was carried out in a volume of 25 μl, with 1 μl of template and 1 μl of each primer (10 nM, nucleotide sequences indicated in **Table 1**) using the Premix Ex Taq version 2.0 kit (Takara, Beijing, China). The procedure was conducted as recommended by the manufacturers with annealing temperature of 50°C and 34 amplification cycles. The amplified products with expected sizes in gel electrophoresis analysis were sequenced by Sangon Biotech company (Shanghai, China). Alignment analyses of the obtained sequences were conducted using the BLAST search engine online (https://blast.ncbi.nlm.nih.gov) for confirmation of the *Rickettsia*-positive samples.

**Table 1:** Primers used for PCR amplification.

Target Gene	Primer Name	Sequence (5′-3′)
*ompA*	Rr190.70p	ATGGCGAATATTTCTCCAAAA
	Rr190.701n	GTTCCGTTAATGGCAGCATCT
	Rr190.602n	AGTGCAGCATTCGCTCCCCCT
*gltA*	CS2d	ATGACCAATGAAAATAATAAT
	CSEndr	CTTATACTCTCTATGTACA
	RpCS.877p	GGGGACCTGCTCACGGCGG
	RpCS.1258n	ATTGCAAAAAGTACAGTGAACA
*rrs*	S1	TGATCCTGGCTCAGAACGAAC
	S2	TAAGGAGGTAATCCAGCCGC
	S3	AACACATGCAAGTCGRACGG
	S4	GGCTGCCTCTTGCGTTAGCT
	16s771r	TAATCTTGCGACCGTACTCC;
	16s565f	TCTTAGATATTAGGAGGAACACCG
*Sca4*	D1f	ATGAGTAAAGACGGTAACCT
	RrD2685R	TTCAGTAGAAGATTTAGTACCAAAT
	RrD1826R	TCTAAATKCTGCTGMATCAAT
	NewRrD1713F	CTCTGAATTAAGCAATGCGG
	RrD749F	TGGTAGCATTAAAAGCTGATGG
	D3069R	TCAGCGTTGTGGAGGGGAAG
	D3072R	TGATCAGCGTTGTGGAGGGG
	D1f	ATGAGTAAAGACGGTAACCT
	D928r	AAGCTATTGCGTCATCTCCG
	D2338f	GATGCAGCGAGTGAGGCAGC
	T5DR1	CTGATAAAGCTGTAGCTGCATTA
	RrD2685R	TTCAGTAGAAGATTTAGTACCAAAT
*ompB*	RompB11F	ACCATAGTAGCMAGTTTTGCAG
	RompB2553R	GAATTTTCAAAAGCAATYGTATCAGT
	RAK1452R	SGTTAACTTKACCGYTTATAACTGT
	RAK1009F	ACATKGTTATACARAGTGYTAATGC
	RompB2553R	GAATTTTCAAAAGCAATYGTATCAGT
	RompB2409F	CCGTAACATTAAACAAACAAGCTG
	RompB4887R	AGAGTACCTTGATGTGCRGTATAYT
	RompB3637R	GAAACGATTACTTCCGGTTACA
	NewRompB3521F	GATAATGCCGATGCAAATTGCAG
	RompB3521F	GATAATGCCAATGCAAATTTCAG
	120-807R	CCTTTTAGATTACCGCCTAA
	120-607F	AATATCGCTGACGGTCAAGGT
	RompB1902R	CCGTCATTTCCAATAACTAACTC
	RR1595F	GCCGGAGTTGTCCAATTATCA
	120-2988R	CCGGCTATACCGCCTGTAGT
	120-2788F	AAACAATAATCAAGGTACTGT
	RompB4224F	ACCAAGATTATAAGAAAGGTGATAA
	120-4346R	CGAAGAAGTAACGCTGACTT

The sequences of *ompB*, *sca4*, *gltA*, 16S ribosomal DNA (*rrs*), and *ompA* in those *Rickettsia*-positive samples were amplified by nested PCR and sequenced. Here, PrimeSTAR GXL Premix Fast, Dye plus kit (Takara) that contains high fidelity enzyme was used for PCR amplification. The total volume and the usage amounts of the templates and primers were the same as above described. The annealing temperatures were set to be 55°C or 60°C as recommended. The primers reported in previous publications were used, with some modifications (**Table 1**) (Regnery et al., 1991; Roux et al., 1996; Roux and Raoult, 2000; Jiang et al., 2005). Long sequences were divided into several short ones for amplification and sequencing. Briefly, *ompB* gene was amplified in four parts, with primer combinations RompB11F & RompB2553R and RompB2409F & RompB4887R in the first rounds, and primer combinations RompB11F & RAK1452R, RAK1009F & RompB2553R, RompB2409F & RompB3637R, and RompB3521F (or NewRompB3521F for strain Huaian-HF) & RompB4887R in the second rounds. *sca4* gene was amplified in two parts, with primer combinations D1f & RrD2685R and RrD749F & D3069R (or D3072R for strain Huaian-HFL) in the first rounds and primer combinations D1f & RrD1826R and NewRrD1713F & D3069R (or D3072R for strain Huaian-HFL) in the second rounds. For *gltA*, the first round was conducted as described above, and the second rounds used primer combinations CS2d & RpCS.1258n and CSEndr & RpCS.877p. The hemi-nested PCR assay targeting partial *ompA* gene (532 bp) was conducted as described previously (Regnery et al., 1991; Roux et al., 1996). The unmentioned primers listed in **Table 1** and the second round primers were used as sequencing primers, and the obtained partial sequences were assembled using SnapGene software (Insightful Science; SnapGene.com). In each reaction, both negative and positive controls (using distilled water or genomic DNA of *R. heilongjiangensis* as templates) were included.

### Phylogenetic analysis

The obtained sequences of *rrs*, *gltA*, *ompA*, *ompB*, and *sca4* genes were first aligned with the corresponding sequences in the GenBank database using the BLAST search engine. Those homologous sequences, especially from available whole genomes, were selected. Phylogenetic analysis based on a concatenated sequence of these five genes was conducted using the maximum likelihood method with the General Time Reversible model and bootstrap value of 1,000 in MEGA 7.0 software.

### SFGR-specific IgG antibody test of human sera

To evaluate the serological prevalence of spotted fever in humans, specific Immunoglobulin G (IgG) antibody to SFGR in human sera was evaluated using a commercial Human SFG-IgG Antibody ELISA kit (Jiangsu Meimian Industrial Co. Ltd., Jiangsu, China) as per the manufacturer’s instruction. According to the recommended criteria, a cutoff value was determined as the mean OD450 of the negative controls plus 0.15. A sample with an OD450 value over the calculated cutoff value was considered positive.

### Statistical analysis

The positive rates of *Rickettsia* in ticks feeding on different animals and the seropositive rates in human serum samples from different areas were statistically analyzed using the continuity-adjusted Chi-square test and Chi-square test, respectively, calculated in Microsoft Excel software (Microsoft Co., Ltd., USA). The OD450 value of the human serum samples from different areas were statistically analyzed using one-way ANOVA with SAS 9.1 software. *P* < 0.05 was considered statistically significant.

### Nucleotide sequence deposition

The obtained nucleotide sequences were deposited into the GenBank database with accession numbers ON600642-ON600663 and ON016525-ON016529.

## Results

### Tick species identification

**Figure 2:**
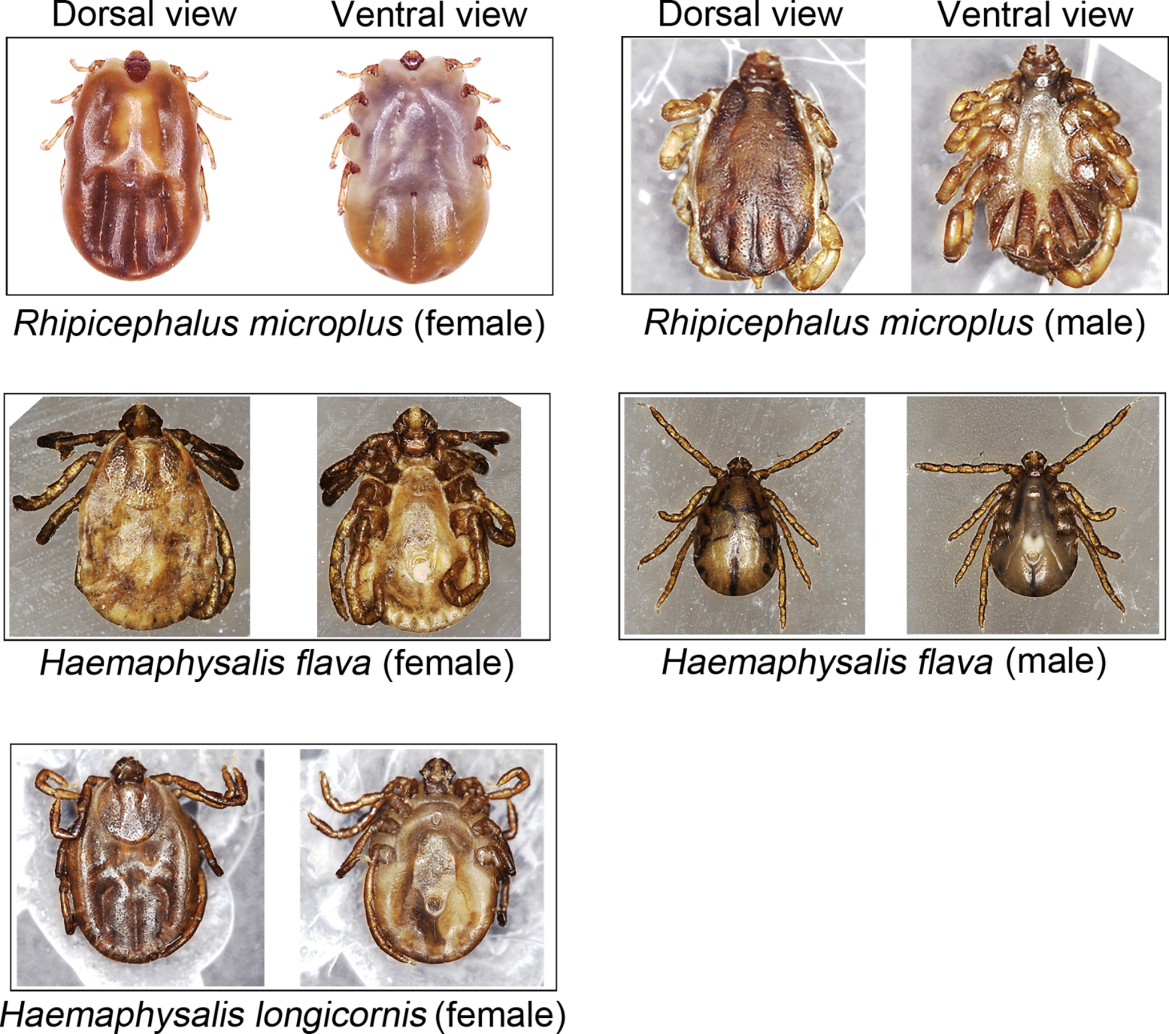
*Haemaphysalis* spp. and *Rhipicephalus microplus* ticks collected in this study.

The ticks were first identified by their morphological features (**Figure 2**) and then confirmed or corrected by molecular method as our previously described (Qi et al., 2022). Overall, 167 (96.5%) of the 173 hedgehog-attached ticks were *H. flava* and the other 6 (3.5%) were *H. longicornis*, whereas 152 (90.5%), 15 (8.9%), and 1 (0.6%) of the 168 bovine-attached ticks were *H. longicornis*, *Rhipicephalus* (*Rh.*) *microplus*, and *H. flava*, respectively.

### Molecular characterization of *Rickettsia* spp.

Overall, three *Rickettsia* spp. strains were identified in the tick samples according to the amplified partial *gltA* gene sequence diversity, namely, Huaian-HF, Huaian-HFL, and Huaian-RM.

**Figure 3:**
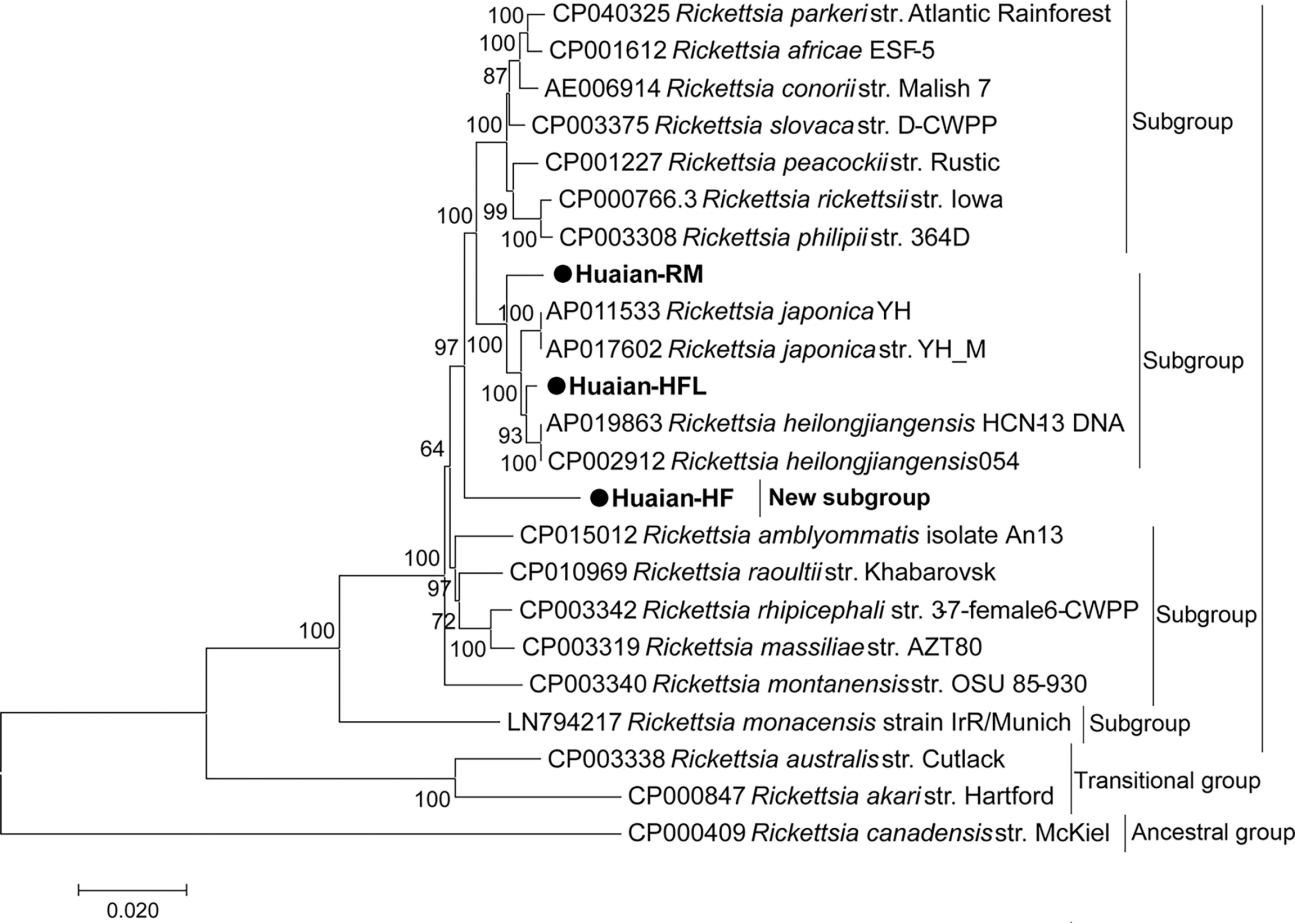
Phylogenetic tree of *Rickettsia* spp. based on a concatenated sequence of *gltA*, *rrs*, *ompA*, *ompB*, and *sca4* gene sequences. The phylogenetic tree was generated with MEGA 7.0 software using the maximum likelihood method with 1,000 replicates for bootstrap testing. Bootstrap values > 50% are shown. The scale bar indicates nucleotide substitutions per site. The GenBank accession numbers of the reference sequences are indicated. The newly obtained sequences are indicated with black dots.

Partial gene sequences of strain Huaian-HF, including *gltA* (1290 bp), *rrs* (1331 bp), *ompA* (575 bp), *ompB* (4807 bp), and *sca4* (3035 bp), were obtained. In BLAST search, those genes shared the highest homology with various validated *Rickettsia* spp. (**Table 2**), which was agreement with the gene sequence-based criteria for novel *Rickettsia* sp. identification established previously (Fournier et al., 2003). In this criteria, a new species in the *Rickettsia* genus should possess no more than one value of nucleotide sequence similarity above 99.9%, 99.8%, 98.8%, 99.2%, and 99.3% to the *gltA*, *rrs*, *ompA*, *ompB*, and *sca4* genes of any validated *Rickettsia* spp., respectively (Fournier et al., 2003). However, we found that the *gltA* gene shared 99.92% identity rate with the corresponding gene of the uncharacterized and unvalidated *Candidatus* R. principis, with a query coverage of 96%, indicating the strain Huaian-HF was likely to belong to the *Candidatus* R. principis. In the phylogenic analysis based on the concatenated sequence of the five genes (**Figure 3**), this strain did not cluster with any validated *Rickettsia* spp. or belong to any previously determined subgroup (Diop et al., 2020). Therefore, we confirmed that *Candidatus* R. principis formed a novel subgroup in the SFG.

**Table 2:** Identity rates of the genes from the identified strains with various validated or unvalidated *Rickettsia* spp.

Strains	Gene	*gltA*	*rrs*	*ompA*	*ompB*	*sca4*
Huaian-HF	Size	1,290 bp	1,331 bp	575 bp	4,807 bp	3,035 bp
	Highest Identity Rate	98.84% to *R. peacockii* strain Rustic; 99.92% to *Candidatus* R. principis (96% coverage)	99.32% to *R. slovaca* strain D-CWPP and *R. rhipicephali* strain HJ#5	95.37% to *R. parkeri* strains Atlantic Rainforest and Black Gap (80% coverage)	94.82% *R. rhipicephali* strain HJ#5	97.79% *R. slovaca* str. D-CWPP
Huaian-HFL	Size	1,289 bp	1,331 bp	632 bp	4,856 bp	2,993 bp
	Highest Identity Rate	99.77% to *R. heilongjiangensis* 054; 99.69% to *R. japonica* YH_M	99.92% and 100% to *R. japonica* YH_M and strain Shandong J71, respectively; 99.85% to *R. heilongjiangensis* 054	98.10% to *R. heilongjiangensis* strain CH8-1; 97.15% to *R. japonica* YH_M; 100% to various *R. japonica* strains Shandong J68 to J75	99.03% to *R. heilongjiangensis* 054; 98.65% to *R. japonica* YH_M	99.63% to *R. heilongjiangensis* 054; 99.50% to *R. japonica* YH_M
Huaian-RM	Size	1,260 bp	1,331 bp	523 bp	4,842 bp	2,660 bp
	Highest Identity Rate	99.60% to *R. heilongjiangensis* 054; 99.52% to *R. japonica* YH_M	99.70% to *R. japonica* YH_M; 99.77% to *Candidatus* R. longicornii isolate GD02	94.87% to *R. heilongjiangensis* 054; 100% to *Candidatus* R. longicornii and *Candidatus* R. jingxinensis isolate Rm1	98% to *R. japonica* strains HH-16 to 18	98.5% to *R. japonica* YH_M
Novel species Criteria	Identity Rates (Fournier et al., 2003)	<99.9%	<99.8	<98.8	<99.2	<99.3

For strain Huaian-HFL, *gltA* (1289 bp), *rrs* (1331 bp), *ompA* (632 bp), *ompB* (4856 bp), and *sca4* (2993 bp) genes shared the highest homology with either *R. heilongjiangensis* or *R. japonica* (**Table 2**). In the phylogenic analysis (**Figure 3**), this strain clustered with *R. heilongjiangensis* and *R. japonica*, belonging to the *R. japonica* subgroup defined previously (Diop et al., 2020). Judged from the phylogenic tree, it is evolutionarily closer to *R. heilongjiangensis* than *R. japonica*. Therefore, we proposed the strain belonged to *R. heilongjiangensis.*

Similar with strain Huaian-HFL, *gltA* (1260 bp), *rrs* (1331 bp), *ompA* (523 bp), *ompB* (4842 bp), and *sca4* (2660 bp) genes of strain Huaian-RM shared the highest homology with validated species of either *R. heilongjiangensis* or *R. japonica* (**Table 2**), with identity rates agreement with the gene sequence-based criteria for a novel species discrimination (Fournier et al., 2003). However, its partial *ompA* gene shared 100% identity rate with *Candidatus* R. longicornii and *Candidatus* R. jingxinensis, indicating that it belonged to *Candidatus* R. longicornii or *Candidatus* R. jingxinensis, although its *rrs* gene shared 99.77% (< 99.8%, the criteria) identity rate with *Candidatus* R. longicornii isolate GD02. It seems that *Candidatus* R. longicornii and *Candidatus* R. jingxinensis belong to the same species. Judged from the phylogenic analysis (**Figure 3**), the strain Huaian-RM also belonged to *R. japonica* subgroup.

### Prevalence of *Rickettsia* spp. in ticks

Overall, 144 (83.2%) of the 173 ticks from hedgehogs and 2 (1.2%) of the 168 ticks from bovine were positive for *Rickettsia* spp., and the significant difference between the two groups existed (*P* = 3.6e-52). *Candidatus* R. principis Huaian-HF (9, 5.2%) and *R. heilongjiangensis* Huaian-HFL (135, 78.0%) were detected in *H. flava* rather than *H. longicornis* ticks from hedgehogs. However, *R. heilongjiangensis* Huaian-HFL (1; 0.6%) and *Candidatus* R. jingxinensis (or *Candidatus* R. longicornii) Huaian-RM (1; 0.6%) were detected in *H. longicornis* and *Rh. microplus* ticks from bovine, respectively.

### Serological prevalence of spotted fever in humans

**Figure 4:**
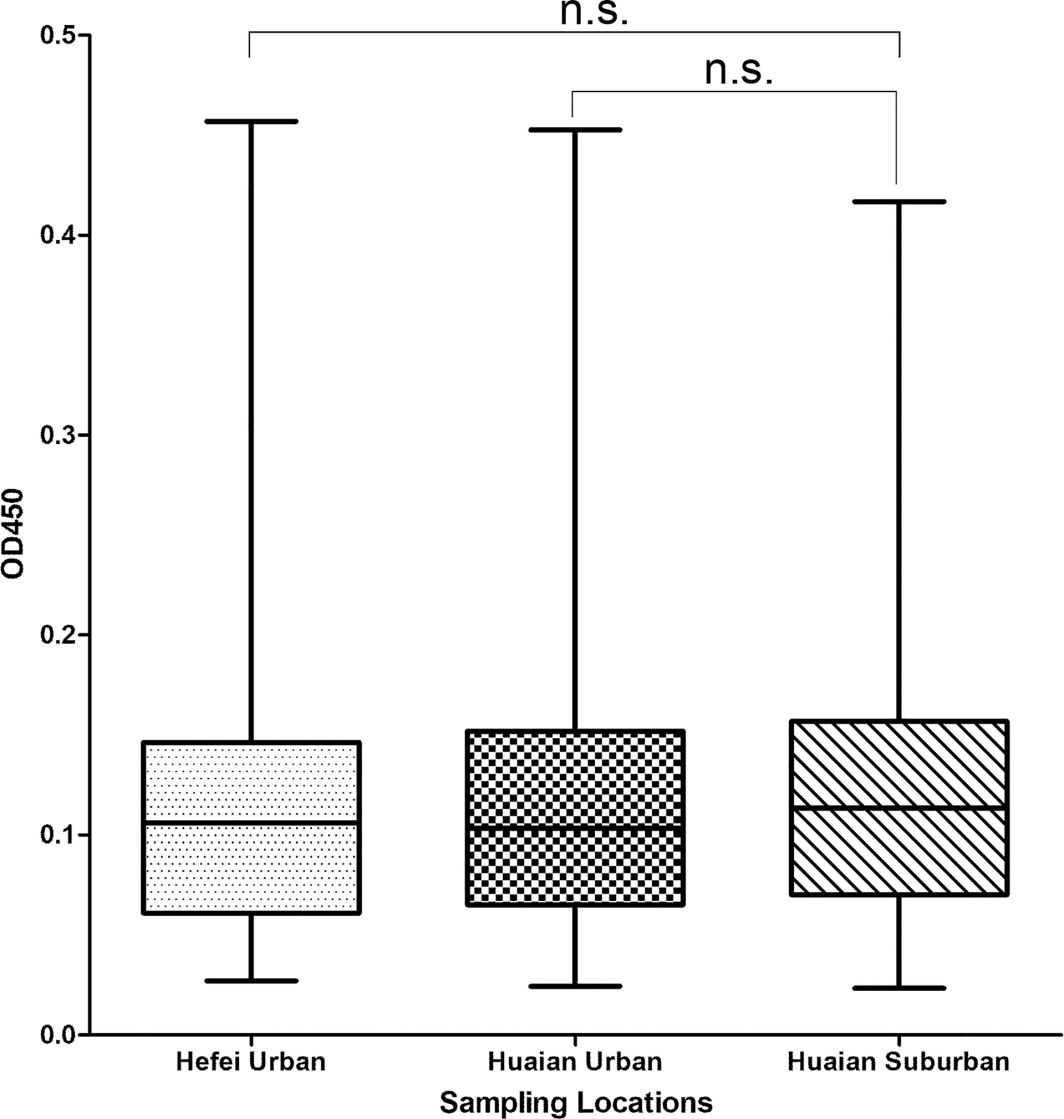
Box and whisker plot of OD450 values of the serum samples detected using ELISA. The box represents 25th to 75th percentile and the mean value (line in the middle of each box), and whisker shows the minimum and maximum values. The sampling locations were indicated. n.s., not significant.

SFGR-specific IgG antibody in human sera samples was detected by ELISA. As a result, 5.57% (18 of 323), 4.85% (13 of 268), and 4.00% (8 of 200) of the tested samples from hospitals in Xuyi county of Huaian city, urban area of Huaian city, and urban area of Hefei city, respectively, were SFGR-specific IgG antibody positive, whereas the differences between them were not significant (*P* > 0.05). In addition, the samples collected from Xuyi county of Huaian city performed a higher mean of OD450 value than those from urban areas of Huaian or Hefei city, whereas no significant difference was observed (**Figure 4**, *P* > 0.05).

## Discussion

In the present study, molecular prevalence of SFGR was investigated in ticks from wild hedgehogs and domestic bovine in Jiangsu province, Eastern China. Surprisingly, the ticks’ *Rickettsia*-positive rates varied significantly, with *Rickettsia*-positive rate for ticks from hedgehogs (83.2%) being significantly higher than that for ticks from bovine (1.2%). We suppose three reasons may attribute to the distinctions. First, hedgehogs may play a role as a “bridge” that transmits the pathogen from infected ticks to uninfected ones. Compared with bovine, hedgehogs are much smaller, and the attached ticks are closer to each other on the inoculation sites of skin, making it easier for pathogen transmission from tick to tick through the circulatory system of the small mammal. Second, in contrast to wild hedgehog-attached ticks, ticks on domestic bovine are regularly removed by bovine owners with insecticides or manually, and pathogens cannot be effectively transmitted between ticks within limited time frame. In this sense, the *Rickettsia*-positive rate of ticks on bovine may reflect the positive rate of free ticks. Third, *Rickettsia* spp. may have their preferred host and different tick species may harbor different pathogens with different positive rates. Nevertheless, investigations on epidemiological data of animal hosts, their attached tick species, and the tick harboring and transmitting *Rickettsia* spp. may benefit the prevention and control strategy development for spotted fever (Chisu et al., 2020; Song et al., 2021; Qi et al., 2022).

In recent years, dozens of novel *Rickettsia* spp. have been identified, such as *Candidatus* R. Thierseensis, *Candidatus* R. uralica, *Candidatus* R. xinyangensis, *Candidatus* R. africaseptentrionalis, *Candidatus* R. mauretanica, *Candidatus* R. jingxinensis, and *Candidatus* R. longicornii (Liu et al., 2016; Jiang et al., 2018; Buysse and Duron, 2020; Li et al., 2020a; Schötta et al., 2020). However, some of them, with only few short partial genes published, have not been validated. The loss of sequence data of critical genes of the unvalidated species typically misleads scientists to mistakenly believe they have identified a new species. For example, *Candidatus* R. thierseensis and *Candidatus* R. uralica are recently found to be genetic variants of one species (Igolkina et al., 2022). Here, in the present study, *Candidatus* R. longicornii and *Candidatus* R. jingxinensis are believed to belong to one species, by sharing 100% identity rate of partial *ompA* gene between their isolates (**Table 2**). Considering *Candidatus* R. jingxinensis was named earlier, we propose *Candidatus* R. jingxinensis as its final species name. The long or near full-length sequences of these phylogenetically important genes were obtained for the first time, and it was phylogenetically characterized as a species of the subgroup around *R. japonica*. Until now, it was only identified in East Asia, including Republic of Korea, and Southwestern and Northern China (Guo et al., 2019; Qin et al., 2019; Kim et al., 2020; Li et al., 2020b; Seo et al., 2020; Jiao et al., 2021b; Park et al., 2021), whereas it was, for the first time, identified in Jiangsu province, Eastern China. Similar to other species (i.e., *R. heilongjiangensis* and *R. japonica*) in this subgroup, *Candidatus* R. jingxinensis was observed to be able to infect humans with clinical characteristics of fever, erythematous rash, and eschar (Kim et al., 2020), indicating a potential threat to public health of the area.

Another uncharacterized species *Candidatus* R. principis was first identified in 2006 by sequencing its *gltA* gene from *H. japonica* ticks in Khabarovsk city, Russia (Mediannikov et al., 2006). Since then, this novel species has been reported to be found in ticks (including *H. japonica*, *H. megaspinosa*, *H. flava*, *H. danieli*, *H. qinghaiensis*, and *I. persulcatus*) in Russian Far East, eastern Hokkaido and Niigata Prefecture (Japan), and Northern and Northwestern China (Chahan et al., 2007; Igolkina et al., 2018; Arai et al., 2021; Okado et al., 2021). This is the first time for *Candidatus* R. principis identified in Eastern China. However, it had not been fully characterized, with limited short gene sequences available except *gltA* gene. Interestingly, according to our molecular characterization and phylogenetic analysis, *Candidatus* R. principis itself was believed to form a novel subgroup of SFGR (**Figures 4**), with low identity rates of its *ompA* and *ompB* genes with those validated *Rickettsia* spp. (**Table 2**). Because very limited studies and evidence available now, its pathogenicity to humans still needs further investigation and surveillance.

In addition, *R. heilongjiangensis* Huaian-HFL was identified in both *H. flava* from hedgehogs and *H. longicornis* from bovine. As the causative agent of Far-eastern spotted fever (FESF), *R. heilongjiangensis*–infected patients show symptoms of fever, chills, headache, dizziness, myalgia, arthralgia, and anorexia, after which most of them show signs of a macular or maculopapular rash and lymphadenopathy near the eschar (Cheng et al., 2016). Previous genome analysis revealed that *R. heilongjiangensis* is closely related to *R. japonica*, with only a small difference in the gene repertoire (Duan et al., 2011; Kasama et al., 2019). Therefore, it is not surprising that the strain was classified as a *R. heilongjiangensis* strain in the present study although some *Rickettsia* spp. strains sharing 100% identity rates in *rrs* or *ompA* gene with it were classified as *R. japonica* (e.g., strains Shandong J68 to J75) previously. In addition, the used genes and the obtained sequence length (partial or whole) for typing must have influenced the classification result variations. Because gene sequences from various isolates and strains of both *Rickettsia* spp. are available, their distinctions may fall outside the criteria for discriminating species as described previously (Fournier et al., 2003). Thus, to reduce controversies over the classification of new strains or isolates in the future, we recommend that whether both species as well as their isolates and strains are actual subspecies of the same *Rickettsia* sp. should be reevaluated and reassessed.

Considering the high pathogenic *Rickettsia*-positive rate in ticks from wild hedgehogs, we supposed people might be exposed to SFGR in the investigated area. On the one hand, living area of hedgehogs typically overlaps with that of humans in (sub) urbans, leading to possible contacts and exposure of people to the pathogens that their parasitic ticks harbor (Ruszkowski et al., 2021; Qi et al., 2022); on the other hand, ticks are both vectors and reservoirs for most SFGR, which may infect wild or domestic animals and humans through tick biting (Socolovschi et al., 2009). Sera samples were collected from the Xuyi County Hospital of Chinese Medicine, which is believed to receive more people living in the rural area around the sampling site than the other two hospitals in urban areas. The preliminary seroepidemiological survey confirmed the higher SFGR-specific seropositive rate and specific IgG antibody level in human sera from the county hospital than those from urban hospitals; however, the differences were not significant. The lack of rigorous screening of serum samples from rural populations and the fact that more urban people now travel to rural areas more often may have contributed to the non-significant difference in results, by masking differentiations. Moreover, the serum collection time may influence the results due to the tick-borne pathogen’s temporal distribution heterogeneity. Nevertheless, the preliminary results indicate the higher historical infection rate of SFGR in the investigated area and potential public health threat posed by the pathogens, their tick hosts, and wild animals.

In conclusion, various *Rickettsia* spp. including *R. heilongjiangensis*, *Candidatus* R. jingxinensis (or *Candidatus* R. longicornii), and *Candidatus* R. principis were identified in ticks feeding on hedgehogs and bovine in Jiangsu province, Eastern China, with the latter two species reported for the first time in this area. Fully genetic characterizations of *Candidatus* R. jingxinensis, and *Candidatus* R. principis were done for the first time, and results indicated that *Candidatus* R. jingxinensis belonged to *R. japonica* subgroup and that *Candidatus* R. principis formed a novel subgroup of SFG. High *Rickettsia*-positive rate was observed in ticks from hedgehogs rather than from bovine, suggesting more attention should be paid to pathogens transmitted or harbored by wild animals and their attached ticks. The preliminary seroepidemiological survey revealed a higher level of SFGR-specific IgG antibody and seropositive rate of spotted fever around the investigated area, indicating that SFGR and their tick hosts, especially from wild animals, pose potential threats to public health in this area. This study provides important epidemiological data for local public health officers in developing strategies for early warning, prevention, and control of the potential *Rickettsia* infections and for local physicians in preparation of suitable testing and treatment needs against potential rickettsioses.

## Data availability statement

The original contributions presented in the study are included in the article/supplementary material. Further inquiries can be directed to the corresponding authors.

## Ethics statement

The studies involving human participants were reviewed and approved by Ethics Committee of Huadong Research Institute for Medicine and Biotechniques. The patients/participants provided their written informed consent to participate in this study. The animal study was reviewed and approved by Ethics Committee of Huadong Research Institute for Medicine and Biotechniques. Written informed consent was obtained from the owners for the participation of their animals in this study.

## Author contributions

Conceptualization: YQi, LA, and WT; data curation: YQi, LA, and JJ; formal analysis: CZ and YM; funding acquisition: WT and YL; resources: DW, PW, GZ, YQin, and CH; methodology: RL and NL; software: JW, RQ, and YL; writing—original draft: YQi; Writing—review and editing: WT and YQi. All authors contributed to the article and approved the submitted version.

## Funding

This study was funded by Medical Science and Technology Projects (19SWAQ04 and 2020QN06357), Jiangsu Social Development Project (BE2017620), and China National Natural Science Foundation (U1602223).

## Conflict of interest

The authors declare that the research was conducted in the absence of any commercial or financial relationships that could be construed as a potential conflict of interest.

## Publisher’s note

All claims expressed in this article are solely those of the authors and do not necessarily represent those of their affiliated organizations, or those of the publisher, the editors and the reviewers. Any product that may be evaluated in this article, or claim that may be made by its manufacturer, is not guaranteed or endorsed by the publisher.

## References

[B1] Arai R. Sato M. Kato M. Aoki J. Nishida A. Watanabe K.. (2021). Spotted fever group rickettsiae (SFGR) detection in ticks following reported human case of Japanese spotted fever in niigata prefecture, Japan. Sci. Rep. 11, 2595. doi: 10.1038/s41598-021-81587-9 33510192 PMC7844008

[B2] Buysse M. Duron O. (2020). Two novel rickettsia species of soft ticks in north Africa: 'Candidatus rickettsia africaseptentrionalis' and 'Candidatus rickettsia mauretanica'. Ticks Tick Borne Dis. 11, 101376. doi: 10.1016/j.ttbdis.2020.101376 32005627

[B3] Chahan B. Jian Z. Jilintai Miyahara K. Tanabe S. Xuan X.. (2007). Detection of DNA closely related to 'Candidatus rickettsia principis' in haemaphysalis danieli recovered from cattle in xinjiang uygur autonomous region area, China. Vet. Parasitol. 144, 184–187. doi: 10.1016/j.vetpar.2006.09.019 17052853

[B4] Cheng C. Fu W. Ju W. Yang L. Xu N. Wang Y. M.. (2016). ). diversity of spotted fever group rickettsia infection in hard ticks from suifenhe, Chinese-Russian border. Ticks Tick Borne Dis. 7, 715–719. doi: 10.1016/j.ttbdis.2016.02.023 26976703

[B5] Chisu V. Loi F. Foxi C. Chessa G. Masu G. Rolesu S.. (2020). Coexistence of tick-borne pathogens in ticks collected from their hosts in Sardinia: An update. Acta Parasitol. 65, 999–1004. doi: 10.1007/s11686-020-00240-z 32557083

[B6] Diop A. El Karkouri K. Raoult D. Fournier P. E. (2020). Genome sequence-based criteria for demarcation and definition of species in the genus rickettsia. Int. J. Syst. Evol. Microbiol. 70, 1738–1750. doi: 10.1099/ijsem.0.003963 31935173

[B7] Duan C. Tong Y. Huang Y. Wang X. Xiong X. Wen B. (2011). Complete genome sequence of rickettsia heilongjiangensis, an emerging tick-transmitted human pathogen. J. Bacteriol 193, 5564–5565. doi: 10.1128/JB.05852-11 21914880 PMC3187448

[B8] Fournier P. E. Dumler J. S. Greub G. Zhang J. Wu Y. (2003). Gene sequence-based criteria for identification of new rickettsia isolates and description of rickettsia heilongjiangensis sp. Nov. J. Clin. Microbiol. 41, 5456–5465. doi: 10.1128/JCM.41.12.5456-5465.2003 14662925 PMC308961

[B9] Guo W. P. Wang Y. H. Lu Q. Xu G. Luo Y. Ni X.. (2019). Molecular detection of spotted fever group rickettsiae in hard ticks, northern China. Transbound Emerg. Dis. 66, 1587–1596. doi: 10.1111/tbed.13184 30920159

[B10] Huang M. Ma J. Jiao J. Li C. Chen L. Zhu Z.. (2021). The epidemic of q fever in 2018 to 2019 in zhuhai city of China determined by metagenomic next-generation sequencing. PLoS Negl. Trop. Dis. 15, e0009520. doi: 10.1371/journal.pntd.0009520 34264939 PMC8282036

[B11] Hu J. Li Z. Hong L. Bao C. Zhang Z. Zhang H.. (2017). Preliminary fast diagnosis of severe fever with thrombocytopenia syndrome with clinical and epidemiological parameters. PLoS One 12, e0180256. doi: 10.1371/journal.pone.0180256 28678811 PMC5497983

[B12] Hu J. Shi C. Li Z. Guo X. Qian Y. Tan W.. (2018). A cluster of cases of severe fever with thrombocytopenia syndrome bunyavirus infection in China 1996: A retrospective serological study. PLoS Negl. Trop. Dis. 12, e0006603. doi: 10.1371/journal.pntd.0006603 29940000 PMC6034904

[B13] Igolkina Y. Rar V. Vysochina N. Ivanov L. Tikunov A. Pukhovskaya N.. (2018). Genetic variability of rickettsia spp. in dermacentor and haemaphysalis ticks from the Russian far East. Ticks Tick Borne Dis. 9, 1594–1603. doi: 10.1016/j.ttbdis.2018.07.015 30121164

[B14] Igolkina Y. Rar V. Yakimenko V. Tikunov A. Tikunova N. (2022). "Candidatus rickettsia uralica" and "Candidatus rickettsia thierseensis" are genetic variants of one species. Ticks Tick Borne Dis. 13, 101933. doi: 10.1016/j.ttbdis.2022.101933 35245854

[B15] Jiang J. An H. Lee J. S. O'guinn M. L. Kim H. C. Chong S. T.. (2018). Molecular characterization of haemaphysalis longicornis-borne rickettsiae, republic of Korea and China. Ticks Tick Borne Dis. 9, 1606–1613. doi: 10.1016/j.ttbdis.2018.07.013 30100386

[B16] Jiang J. Blair P. J. Felices V. Moron C. Cespedes M. Anaya E.. (2005). Phylogenetic analysis of a novel molecular isolate of spotted fever group rickettsiae from northern Peru: Candidatus rickettsia andeanae. Ann. N Y Acad. Sci. 1063, 337–342. doi: 10.1196/annals.1355.054 16481537

[B17] Jiao J. Lu Z. Yu Y. Ou Y. Fu M. Zhao Y.. (2021a). Identification of tick-borne pathogens by metagenomic next-generation sequencing in dermacentor nuttalli and ixodes persulcatus in inner Mongolia, China. Parasit Vectors 14, 287. doi: 10.1186/s13071-021-04740-3 34044867 PMC8161991

[B18] Jiao J. Zhang J. He P. Ouyang X. Yu Y. Wen B.. (2021b). Identification of tick-borne pathogens and genotyping of coxiella burnetii in rhipicephalus microplus in yunnan province, China. Front. Microbiol. 12, 736484. doi: 10.3389/fmicb.2021.736484 34621258 PMC8491607

[B19] Kasama K. Fujita H. Yamamoto S. Ooka T. Gotoh Y. Ogura Y.. (2019). Genomic features of rickettsia heilongjiangensis revealed by intraspecies comparison and detailed comparison with rickettsia japonica. Front. Microbiol. 10, 2787. doi: 10.3389/fmicb.2019.02787 31866968 PMC6908463

[B20] Kim Y. S. Kim J. Choi Y. J. Park H. J. Jang W. J. (2020). Molecular genetic analysis and clinical characterization of rickettsia species isolated from the republic of Korea in 2017. Transbound Emerg. Dis. 67, 1447–1452. doi: 10.1111/tbed.13525 32090496

[B21] Liang S. Bao C. Zhou M. Hu J. Tang F. Guo X.. (2014). Seroprevalence and risk factors for severe fever with thrombocytopenia syndrome virus infection in jiangsu province, China 2011. Am. J. Trop. Med. Hyg 90, 256–259. doi: 10.4269/ajtmh.13-0423 24343883 PMC3919226

[B22] Li J. Kelly P. Guo W. Zhang J. Yang Y. Liu W.. (2020b). Molecular detection of rickettsia, hepatozoon, ehrlichia and SFTSV in goat ticks. Vet. Parasitol. Reg. Stud. Rep. 20, 100407. doi: 10.1016/j.vprsr.2020.100407 32448525

[B23] Li H. Li X. M. Du J. Zhang X. A. Cui N. Yang Z. D.. (2020a). Candidatus rickettsia xinyangensis as cause of spotted fever group rickettsiosis, xinyang, China 2015. Emerg. Infect. Dis. 26, 985–988. doi: 10.3201/eid2605.170294 32310072 PMC7181907

[B24] Liu H. Li Q. Zhang X. Li Z. Wang Z. Song M.. (2016). Characterization of rickettsiae in ticks in northeastern China. Parasit Vectors 9, 498. doi: 10.1186/s13071-016-1764-2 27623998 PMC5022169

[B25] Lu M. Li F. Liao Y. Shen J. J. Xu J. M. Chen Y. Z.. (2019). Epidemiology and diversity of rickettsiales bacteria in humans and animals in jiangsu and Jiangxi provinces, China. Sci. Rep. 9, 13176. doi: 10.1038/s41598-019-49059-3 31511528 PMC6739303

[B26] Mediannikov O. Sidelnikov Y. Ivanov L. Fournier P. E. Tarasevich I. Raoult D. (2006). Far Eastern tick-borne rickettsiosis: Identification of two new cases and tick vector. Ann. N Y Acad. Sci. 1078, 80–88. doi: 10.1196/annals.1374.010 17114683

[B27] Merhej V. Angelakis E. Socolovschi C. Raoult D. (2014). Genotyping, evolution and epidemiological findings of rickettsia species. Infect. Genet. Evol. 25, 122–137. doi: 10.1016/j.meegid.2014.03.014 24662440

[B28] Merhej V. Raoult D. (2011). Rickettsial evolution in the light of comparative genomics. Biol. Rev. Camb Philos. Soc. 86, 379–405. doi: 10.1111/j.1469-185X.2010.00151.x 20716256

[B29] Okado K. Adjou Moumouni P. F. Lee S. H. Sivakumar T. Yokoyama N. Fujisaki K.. (2021). Molecular detection of borrelia burgdorferi (Sensu lato) and rickettsia spp. in hard ticks distributed in tokachi district, Eastern Hokkaido, Japan. Curr. Res. Parasitol. Vector Borne Dis. 1, 100059. doi: 10.1016/j.crpvbd.2021.100059 35284860 PMC8906132

[B30] Park H. J. Kim J. Choi Y. J. Kim H. C. Klein T. A. Chong S. T.. (2021). Tick-borne rickettsiae in Midwestern region of republic of Korea. Acta Trop. 215, 105794. doi: 10.1016/j.actatropica.2020.105794 33310079

[B31] Parola P. Paddock C. D. Socolovschi C. Labruna M. B. Mediannikov O. Kernif T.. (2013). Update on tick-borne rickettsioses around the world: A geographic approach. Clin. Microbiol. Rev. 26, 657–702. doi: 10.1128/CMR.00032-13 24092850 PMC3811236

[B32] Parola P. Raoult D. (2001). Tick-borne bacterial diseases emerging in Europe. Clin. Microbiol. Infect. 7, 80–83. doi: 10.1046/j.1469-0691.2001.00200.x 11298147

[B33] Qi Y. Ai L. Zhu C. Lu Y. Lv R. Mao Y.. (2022). Co-Existence of multiple anaplasma species and variants in ticks feeding on hedgehogs or cattle poses potential threats of anaplasmosis to humans and livestock in the Eastern China. Front. Microbiol. 13, 913650. doi: 10.3389/fmicb.2022.913650 35756069 PMC9226643

[B34] Qin X. R. Han H. J. Han F. J. Zhao F. M. Zhang Z. T. Xue Z. F.. (2019). Rickettsia japonica and novel rickettsia species in ticks, China. Emerg. Infect. Dis. 25, 992–995. doi: 10.3201/eid2505.171745 31002060 PMC6478201

[B35] Regnery R. L. Spruill C. L. Plikaytis B. D. (1991). Genotypic identification of rickettsiae and estimation of intraspecies sequence divergence for portions of two rickettsial genes. J. Bacteriol 173, 1576–1589. doi: 10.1128/jb.173.5.1576-1589.1991 1671856 PMC207306

[B36] Roux V. Fournier P. E. Raoult D. (1996). Differentiation of spotted fever group rickettsiae by sequencing and analysis of restriction fragment length polymorphism of PCR-amplified DNA of the gene encoding the protein rompa. J. Clin. Microbiol. 34, 2058–2065. doi: 10.1128/jcm.34.9.2058-2065.1996 8862558 PMC229190

[B37] Roux V. Raoult D. (2000). Phylogenetic analysis of members of the genus rickettsia using the gene encoding the outer-membrane protein rompb (Ompb). Int. J. Syst. Evol. Microbiol. 50 Pt 4, 1449–1455. doi: 10.1099/00207713-50-4-1449 10939649

[B38] Ruszkowski J. J. Hetman M. Turlewicz-Podbielska H. Pomorska-Mól M. (2021). Hedgehogs as a potential source of zoonotic pathogens-a review and an update of knowledge. Anim. (Basel) 11, 1754. doi: 10.3390/ani11061754 PMC8230866 34208276

[B39] Schötta A. M. Wijnveld M. Höss D. Stanek G. Stockinger H. Markowicz M. (2020). Identification and characterization of "Candidatus rickettsia thierseensis", a novel spotted fever group rickettsia species detected in Austria. Microorganisms 8, 1670. doi: 10.3390/microorganisms8111670 PMC7692616 33126449

[B40] Seo M. G. Kwon O. D. Kwak D. (2020). Molecular and phylogenetic analysis of tick-borne pathogens in ticks parasitizing native Korean goats (Capra hircus coreanae) in south Korea. Pathogens 9, 71. doi: 10.3390/pathogens9020071 PMC7168648 31973172

[B41] Socolovschi C. Mediannikov O. Raoult D. Parola P. (2009). The relationship between spotted fever group rickettsiae and ixodid ticks. Vet. Res. 40, 34. doi: 10.1051/vetres/2009017 19358804 PMC2695030

[B42] Song K. Ji Y. Sun S. Yue X. Wang C. Luo T. (2021). Bacterial microbiota in unfed ticks (Dermacentor nuttalli) from xinjiang detected through 16s rDNA amplicon sequencing and culturomics. Zoonoses. 1, 1–11. doi: 10.15212/ZOONOSES-2021-0007

[B43] Wang Q. Guo W. B. Pan Y. S. Jiang B. G. Du C. H. Que T. C.. (2021). Detection of novel spotted fever group rickettsiae (Rickettsiales: Rickettsiaceae) in ticks (Acari: Ixodidae) in southwestern China. J. Med. Entomol 58, 1363–1369. doi: 10.1093/jme/tjaa294 33399212

[B44] Yu P. F. Niu Q. L. Liu Z. J. Yang J. F. Chen Z. Guan G. Q.. (2016). Molecular epidemiological surveillance to assess emergence and re-emergence of tick-borne infections in tick samples from China evaluated by nested PCRs. Acta Trop. 158, 181–188. doi: 10.1016/j.actatropica.2016.02.027 26943995

[B45] Zhang D. Sun C. Yu H. Li J. Liu W. Li Z.. (2019). Environmental risk factors and geographic distribution of severe fever with thrombocytopenia syndrome in jiangsu province, China. Vector Borne Zoonotic Dis. 19, 758–766. doi: 10.1089/vbz.2018.2425 30994412

